# Effectiveness of a Double Air-Cushioned Shoe Compared with Physiotherapy in the Treatment of Plantar Fasciitis

**DOI:** 10.1155/2020/9468302

**Published:** 2020-04-02

**Authors:** S. S. Eun, S. Chachan, S. H. Lee

**Affiliations:** ^1^Department of Orthopedic Surgery, Wooridul Spine Hospital, Gangnam, Seoul, Republic of Korea; ^2^Department of Neurosurgery, Wooridul Spine Hospital, Gangnam, Seoul, Republic of Korea

## Abstract

**Objective:**

This study was aimed at comparing the plantar fasciitis treatment effect of a double air-cushioned shoe to that of physiotherapy combined with ESWT.

**Methods:**

Retrospective chart review of 40 patients diagnosed with plantar fasciitis was performed. Group 1 wore a double air-cushioned shoe for 2 months, and group 2 underwent physiotherapy with ESWT once/week over a 4-week period. The foot function index (FFI) score was obtained at the initial visit, 1 month, and 2 months.

**Results:**

There were 25 patients in group 1 and 15 patients in group 2. The pretreatment FFI was 62.6 for group 1 and 50 for group 2. The 1-month posttreatment FFI was 45.6 for group 1 and 35.7 for group 2. The 2-month posttreatment FFI was 35 for group 1 and 43.1 for group 2. In both groups 1 and 2, follow-up FFIs were significantly improved from the initial FFI (*p* < 0.05) and there were no significant differences between two groups (*p* > 0.05).

**Conclusions:**

The double air-cushioned shoe can be considered an alternative treatment option for noninvasive treatment of early-stage plantar fasciitis.

## 1. Introduction

Plantar fasciitis (PF), a degenerative-inflammatory foot disorder, is the most common cause of heel pain [1–4]. The management gamut encompasses lifestyle modifications, physiotherapy, orthotic use, analgesics, local injections of steroids/platelet-rich plasma (PRP), extracorporeal shock wave therapy (ESWT), and if nothing works then surgery [1–4]. The reported success rate with conservative treatment methods is high (nearly 80%) [1–4]. ESWT improves symptoms by initiating inflammatory response with secretion of growth factors or nitrous oxide and revitalizes tissues by angiogenesis [5, 6].

There is modest evidence regarding utility of foot orthosis in improving plantar fasciitis-related pain [3, 7–12]. A number of theories have been put forward to explain foot orthosis utility like fall in peak heel pressures, reduction in plantar fascia strain, and modified tissue loading [5–10]. Likewise, a number of plantar fasciitis-specific foot orthoses are available, without any proven superiority of one over another [3, 7–9, 11].

**Figure 1:**
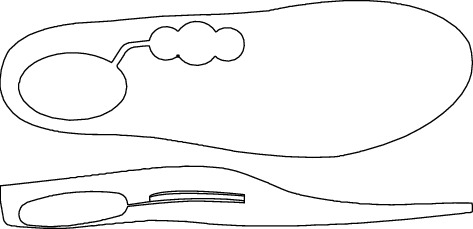
Double air-cushioned insole viewed from above and from the side. Two interconnected heel and arch air bags.

**Figure 2:**
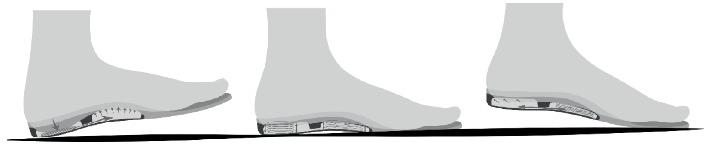
Mechanism of the double air-cushioned shoe during walking. During heel strike, midstance, and toe off phases, air moves from the heel air bag to the arch air bag, providing dynamic support.

Air flow insole (Young Chang Eco Co., Ltd., Busan) is a newly developed shoe insert, which results in a frequent massaging effect and dynamic balancing of foot. It incorporates two interconnected air bags (heel air bag and arch air bag) (Figure 1). During a heel strike phase, the pressure on the heel air bag increases which results in the movement of air to the arch air bag. This supports the medial arch of the foot during the early flat foot phase (Figure 2). In the late flat foot phase, the increased pressure in the medial arch pushes air to the heel air bag. This walking-related to-and-fro air movement helps lower plantar pressure and strain and also provides dynamic support to foot structures.

Due to high prevalence of PF and understandable preference of patients for noninvasive treatment methods, it is paramount to explore and find pertinent options. The current study was to compare the efficacy of a double air-cushioned shoe to that of physiotherapy combined with ESWT in the noninvasive management of PF.

## 2. Patients and Methods

After approval by the institutional review board (IRB; 2016-05-WSH-008), the retrospective chart review was conducted at the orthopedics department of a specialty hospital. Inclusion criteria were set as history of nontreated plantar fasciitis-related heel pain of less than 6-month duration. Patients with more than 6 months of pain, those who have previously received treatment, and those with calcaneus, talus, and metatarsal bone fracture were specifically excluded. Also, patients with comorbidities like lower limb neurovasculopathic conditions, ankle/knee/hip arthrosis, anatomical foot/lower limb deformities, and any other conditions affecting full ambulation were excluded. A total of 40 patients were included. Age ranges from 25 to 77 years. All patients had unilateral symptoms except for three who had bilateral heel pain. Group 1 (shoe) had 25 patients who were asked to wear double air-cushioned shoes for 2 months, whereas group 2 (physiotherapy) had 15 patients who underwent physiotherapy with ESWT once per week over a four-week period. All patients did not undergo any other treatment modalities like injections or oral analgesics. In group 1, the patients were advised to wear the shoes as often as possible. In group 2, physiotherapy and massage were performed for an hour by a physiotherapist with plantar fascia-specific exercises. Approximately 10 min of ESWT (Masterpuls MP200, Storz Medical, Tagerwilen) was instituted with energy of 2 bars by 15,000 pulses. Group 2 patients were instructed to perform stretching, including plantar-specific stretching, unilateral heel raise, and Achilles stretching 10 times with three sessions per day for a 2-month period.

Since PF is associated with both pain and functional decline, the authors preferred the foot function index (FFI) over pain scores to quantify clinical results [4, 13, 14]. The Korean language version of FFI was used [15]. The scores were obtained at the initial visit, 1 month, and 2 months. Self-reported questionnaires were filled in by patients and collected in the outpatient clinic. The Korean version of FFI is a translation of the revised version of FFI, which has 18 questions and a score range from 0 (no pain) to 9 (worst pain imaginable) [15]. The sum of these scores was then expressed as a percentage of the maximum possible score, and the resulting overall score ranged from 0 to 100. Each patient's body mass index (BMI) was also obtained [16].

### 2.1. Statistical Analysis

The statistical analysis was performed using the SPSSWIN 23.0 program (SPSS, Chicago, Illinois, USA), and all results were analyzed at *p* < 0.05 significant level. *T*-test and parametric chi-square tests were performed to see intergroup differences in age, gender, BMI, and initial FFI scores. The repeated measures ANOVA test was used to statistically evaluate the intergroup (shoe and physiotherapy) differences over different periods of time (initial FFI, one-month FFI, and two-month FFI). Multiple regression analysis was used to determine the effects of BMI, age, and gender on FFI.

## 3. Results

**Figure 3:**
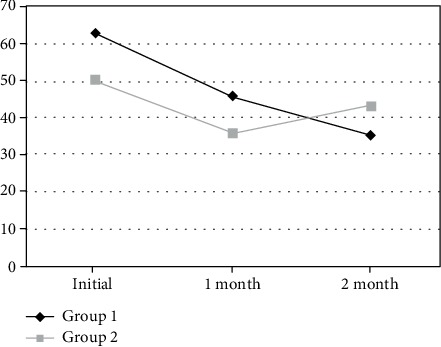
FFI comparison of the shoe (group 1) and physiotherapy (group 2) groups.

As per the *T*-test and chi-square test, the physiotherapy group was insignificantly more aged than the shoe group: 53.6 and 51.2, respectively (*p* > 0.05) (Table 1). No significant difference was found in gender distribution between the shoe and physiotherapy groups (*p* < 0.05). The shoe group showed insignificantly higher initial FFI than the physiotherapy group: 62.6 and 50, respectively (*p* > 0.05). No significant difference was found in BMI between the physiotherapy (24.74) and shoe (24.6) groups (*p* > 0.05). The mean follow-up in both groups was 8 weeks. The FFI improved at 1 month and declined at 2 months in the physiotherapy group: 50.1 at 0 month, 35.7 at 1 month, and 43.1 at 2 months. However, in the shoe group, FFI continuously improved with time and was lowest at 2 months in the air flow insole group: 62.6 at 0 month, 45.6 at 1 month, and 35.0 at 2 months (Figure 3). The repeated measures ANOVA between the FFI scores of two groups (air flow insole and physiotherapy) at different follow-up periods did not find any significant intergroup difference (*F* = 0.607, *p* = 0.441) (Table 2). However, there were significant differences among intragroup FFI scores at different follow-up periods (*F* = 28.2, *p* = 0.001). The interaction effects based on the combination of times and groups also differed significantly (*F* = 10.01, *p* = 0.01). These results confirmed that in both the groups, the 1-month and 2-month follow-up FFIs were significantly improved from the initial FFI. The multiple regression analysis found no significant effects of BMI and age on FFI (Table 3). Although gender was found to have a positive effect on FFI scores (regression coefficient *β* = 0.284), it was statistically insignificant (*t* = 1.71, *p* = 0.095).

## 4. Discussion

The double air-cushioned shoe showed a similar treatment effect to 4 times physiotherapy combined with ESWT in a month over a 2-month period. The shoe group showed more improvement in FFI scores at the 2-month follow-up (statistically insignificant). One reason for this observation may be the continued effect of the shoe since it was worn for the whole period of the observation. Another observation of this study was cost implications of both treatment methods. While the overall cost of four physiotherapy (including ESWT) sessions was 400$, the shoe price was 200$ in South Korea. A weekly physiotherapy treatment with ESWT for a month was our hospital's protocols that patients are satisfied with the treatment effect and the costs.

The double air-cushioned insole has two actions; first, it helps provide cushion to the heel, and second, it raises the medial arch and extends support. On heel strike, the pressure of the heel pushes air to the arch side cushion to a patient's specific arch height. On midstance, the arch is supported by an enlarged air cushion, and shock is also absorbed in the heel side. Dynamic balance is maintained between the two air cushions. This evenly redistributes weight-bearing pressure to the entire plantar surface and takes load off the fascial attachment.

Previous randomized controlled trials suggest that plantar fascia-specific stretching and a shoe insert are effective in the treatment of plantar fasciitis [3, 4, 14, 17]. Lee et al. reported in their meta-analysis study that orthoses provide short-, intermediate-, and long-term benefits for decreasing pain and improving function in plantar fasciitis [18]. Therapeutic footwear is an important tool in the nonsurgical treatment of the plantar fasciitis. This orthosis redistributes and equalizes plantar pressures. Therefore, the entire plantar surface of the foot participates in the weight-bearing process [19]. Orthosis resists depression of the foot's arch during weight bearing, thereby decreasing tension in the plantar aponeurosis, and mechanical relief prevents further trauma to the area and allows the healing process to take its natural course [3, 7, 9]. Rigid orthoses have better support with a less cushioning effect. Soft polyethylene foam has better pressure distribution characteristics when first applied, but the exposure to repeated pressures causes the bottom to thin out more rapidly, limiting its cushioning effect [3, 7, 9, 11]. The double air-cushioned shoe can have a semirigid orthosis property.

Physiotherapy treatments, including exercises and stretching, can be an excellent method in providing targeted and progressive levels of strain to injured soft tissue, which may help appropriate remodeling [1, 2, 20]. Previous studies reported that a significant proportion of patients continue to have symptoms [1, 2, 20, 21]. The limitation of exercise is that no data are obtained for adherence and quality of home-based exercise. In contrast, wearing a shoe requires less effort, with a long-lasting effect, as long as patients keep wearing shoes.

ESWT is reported to be effective in treating plantar fasciitis [1, 4, 22]. ESWT produces force through cavitation. The strong power exerted in plantar fascia by moving the bubble mechanism causes mechanical tissue disruption. The repair of mechanical tissue disruption is a theoretical basis for the neovascularization process and subsequent pain relief after ESWT [1, 4, 22].

The FFI is one of the most frequently used self-reported questionnaires in the evaluation of foot disorders [13–15]. The FFI was developed to measure foot problems on function for pain and disability [13–15]. Huh et al. reported the Korean version of the FFI, and it showed a satisfactory psychometric property [15].

The small sample size, short-term follow-up, and lack of any measure to quantify patient compliance with retrospective review are limitations to this study. Because many patients still have pain after exercise or orthosis treatment, a long-term follow-up with a larger number of patients may be necessary [1–4]. Nonetheless, this study to compare the short-term therapeutic effect of newly designed orthosis to that of physiotherapy has a successful result.

## 5. Conclusion

The double air-cushioned shoe can be considered an alternative option for noninvasive treatment of early-stage plantar fasciitis.

## Figures and Tables

**Table 1:** Intergroup age, gender, BMI, and initial FFI score analysis.

	Group				*t*/*x*^2^ (*p*)
	Air flow insole		Physiotherapy		
	*M*/*n*	SD (%)	*M*/*n*	SD (%)	
Age	51.24	13.2	53.67	15.9	0.521 (0.606)
Sex					
M	10	(40.0)	6	(40.0)	0.000 (1.000)
F	15	(60.0)	9	(60.0)	
Initial FFI	62.68	21.5	50.07	17.9	1.904 (0.065)
BMI	24.63	3.1	24.74	4.4	0.096 (0.924)

**Table 2:** Repeated measures ANOVA of FFI scores between the groups and among times.

Source	Type III sum of squares	df	Mean square	*F*	Sig.
Between-subjects effects					
Intercept	231472.080	1	231472.080	212.056	0.000
Group	662.480	1	662.480	0.607	0.441
Error	41479.387	38	1091.563		
Tests of within-subjects contrasts					
Factor	5624.670	1	5624.670	28.205^∗∗∗^	0.000
Factor∗group	1996.920	1	1996.920	10.014^∗∗^	0.003
Error (factor)	7577.880	38	199.418		

^∗^ *p* < 0.05, ^∗∗^*p* < 0.01, and ^∗∗∗^*p* < 0.001.

**Table 3:** Multiple regression analysis of effects of age, gender, and BMI on FFI scores.

	Unstandardized coefficients		Standardized coefficients	*t*	Sig.	Collinearity statistics	
	*B*	Std. error	Beta			Tolerance	VIF
(Constant)	40.967	27.005		1.517	0.138		
BMI	0.208	0.992	0.036	0.210	0.835	0.886	1.129
Age	-0.141	0.255	-0.095	-0.551	0.585	0.863	1.158
Sex	11.993	7.002	0.284	1.713	0.095	0.934	1.071

*R* square (adjusted *R* square) = 0.076 (0.001), *F* = 0.993 (0.407).

## Data Availability

Most data are reported in this article.

## Abbreviations

PF: Plantar fasciitis
PRP: Platelet-rich plasma
ESWT: Extracorporeal shock wave therapy
IRB: Institutional review board
FFI: Foot function index
BMI: Body mass index.
